# Reconstruction for labial adhesion in postmenopausal woman using vulvoperineal flap

**DOI:** 10.1080/23320885.2019.1602474

**Published:** 2019-04-23

**Authors:** Marika Takimoto, Tomoya Sato, Shigeru Ichioka

**Affiliations:** Department of Plastic and Reconstructive Surgery, Saitama Medical University, Saitama, Japan

**Keywords:** labial adhesion, labial fusion, perineal pain, postmenopausal woman, vulvoperineal flap

## Abstract

We report the case of an 86-year-old postmenopausal woman with severe labial adhesion. The adhesion overlying the vestibule was manually separated and the skin defect was covered with bilateral vulvoperineal flaps. Reconstruction using the vulvoperineal flap enabled to prevent recurrence by covering with normal skin tissue.

## Case report

### History of the present illness

An 86-year-old postmenopausal woman (gravida 2 para 2) presented with a complaint of perineal pain and dysuria. She experienced menopause at the age of 33 years due to a hysterectomy, and her last sexual activity was at the age of 43 years. The patient’s medical history was unremarkable. She first consulted a gynaecologist who later referred her to the Plastic and Reconstructive Surgery Department at the university hospital considering her concern about an impending urinary obstruction.

### Physical findings

**Figure 1.**
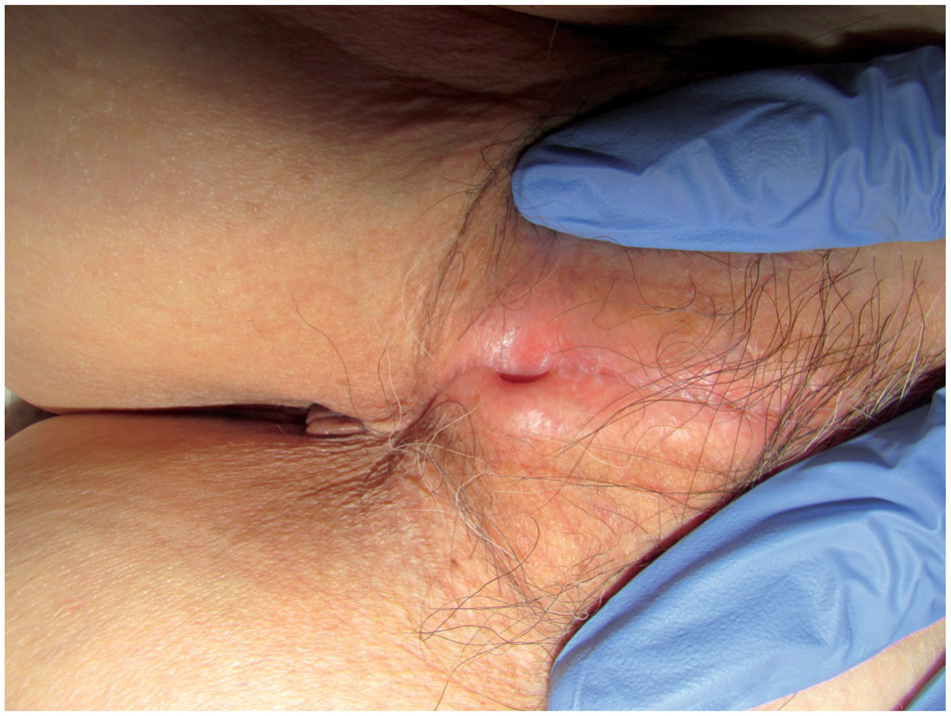
Both labia majora are extensively fused. There are two pinholes, but we could not identify the clitoris, vestibule, labia minora, and external urethral meatus.

Physical examination revealed that both labia majora had extensively fused together and the mucosa was atrophic. There were two pinholes each with a 3 mm diameter: one at the fused midline and the other directly above the vaginal orifice. We could not identify the clitoris, vestibule, labia minora, and external urethral meatus (Figure 1).

Erythema and local warmth were observed in the bilateral labia majora. However, mass or white lesions and hardening of the skin were not observed. Urination was performed by urinating through the pinholes. Although manual separation of the adhesion without local anaesthesia was attempted, it was unsuccessful due to severe pain. Virology tests were positive for human herpes virus type 1 and type 2 antibodies, and endocrine tests showed a marked decrease in oestradiol levels (5.0 pg/mL). In addition, MRI revealed no abnormality except for the absence of a uterus due to prior hysterectomy.

### Operation

With the patient in the lithotomy position and under general anaesthesia, incisions were made on the skin–mucosal boundary of the bilateral labia majora. The clitoris, labia minora, vestibule, and urethral meatus could only be observed by recognising the two pinholes (diameter, 3 mm) along the fused midline. While performing dissection through a small hole just anterior to the vagina and toward the pubic bone, the labial adhesion could be easily separated. Mucosa was removed using VERSAJET from the bilateral labia majora, labia minora, clitoral surface, and vaginal introitus. When the urethral meatus was identifiable, a Foley catheter was inserted into the bladder.

**Figure 2.**
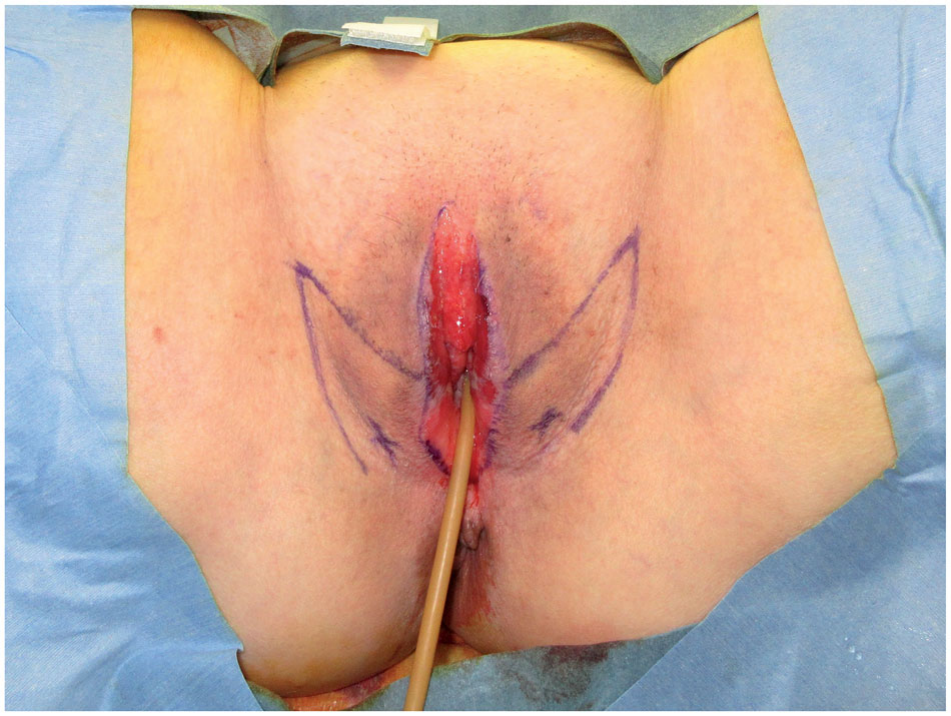
A 3 × 7 cm vulvoperineal flap that incorporates the perforator from the superficial external pudendal artery as the vascular pedicle was designed.

**Figure 3.**
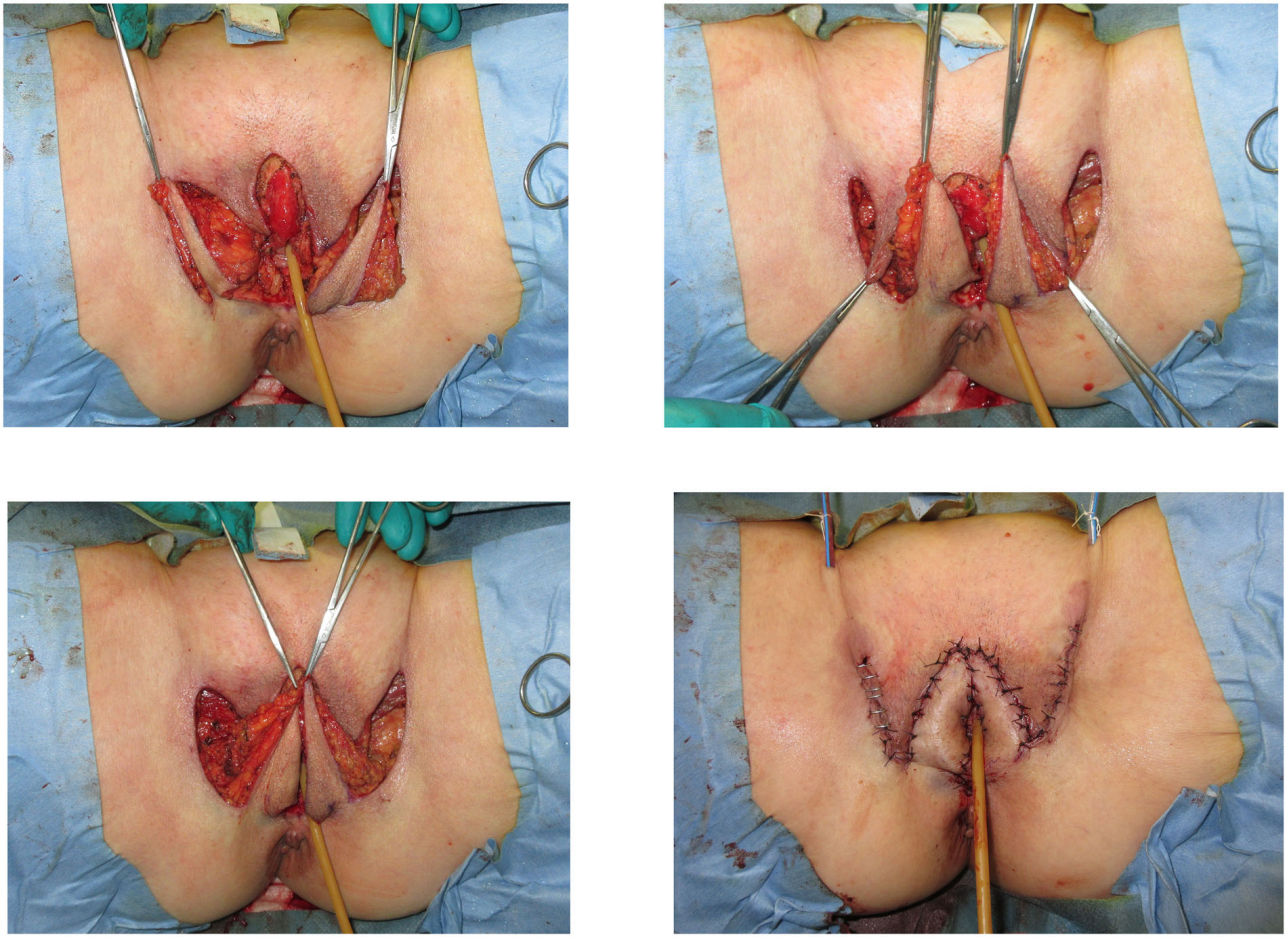
Bilateral 3 × 7 cm vulvoperineal flaps incorporating the perforator from the superficial external pudendal artery as the vascular pedicle were designed.

Bilateral 3 × 7 cm vulvoperineal flaps incorporating the perforator from the superficial external pudendal artery as the vascular pedicle were designed (Figure 2). A skin incision was made along the design from the lateral to medial regions, including the underlying fascia of the adductor longus muscle to ensure the inclusion of the vascular pedicle within the flap (Figure 3). The flap was transferred to the skin defect on the labia majora and clitoris, forming the edge of the external vestibule. Drainage tubes (15 Fr) were subcutaneously inserted on both sides.

### Pathological findings

Infiltration of neutrophils was observed in the epithelium. Inflammatory cell infiltration mainly included lymphocytes, and plasma cells were observed in the dermis. Bacteria were suspended in a mucus-like matter, and neither neoplastic nor malignant findings were observed.

### Postoperative care

**Figure 4.**
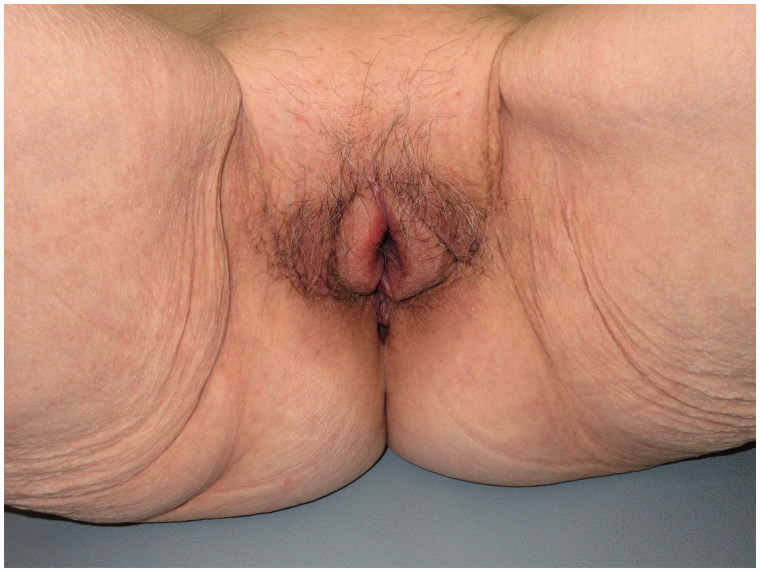
The patient’s urinary function is normal with no recurrence of labial adhesion.

To prevent readhesion and optimise flap perfusion, we instructed the patient to undertake bed rest with the hips in abduction. Temporary oedema of the flap was observed on postoperative day 2, but it gradually resolved by postoperative day 6 due to continuous hip abduction. Bilateral flap drains and the Foley catheter were removed on postoperative day 7 when ambulation started. Further, sutures were removed on postoperative day 14 with no findings of tightness or pain. The patient was discharged on postoperative day 25, and she underwent rehabilitation during her admission until discharged. Notably, no recurrence was observed after postoperative 18 months (Figure 4).

## Discussion

We report the case of an 86-year-old postmenopausal woman with severe labial adhesion reconstructed using a vulvoperineal flap. Furthermore, we succeeded in preventing recurrence for almost a year postoperatively.

Labial adhesion is prevalent in girls before puberty or in postmenopausal women. In infants, it is often caused by local contamination and irritation due to diapers, resulting in vulvitis [1]. As observed in adrenocortical hyperplasia and adrenogenital syndrome, congenital abnormalities of the external genitalia secondary to an endocrine abnormality can exist. However, labial adhesion in postmenopausal women is an acquired condition commonly caused as a result of poor hygiene, eczema, lichen planus, seborrhoeic dermatitis, local trauma, and recurrent urinary tract infection and is defined as complete or partial fusion of both labia minora along the midline. Moreover, low oestrogen levels and absence of sexual activity contribute to the formation of adhesions.

The physical findings of labial adhesion are typical. The vaginal orifice, clitoris, and urethral meatus are not visible, and the labia minora are sealed. A thin central line of adherence running from the clitoris to the posterior fourchette is pathognomonic. A small single aperture is commonly found in the sub-clitoral area through which micturition can occur [2]. This disorder is relatively unusual in women of reproductive age. The extent of adhesion and type of cases vary from simple linear adhesions that can be manually separated to extensive fusion of the labia majora and labia minora.

The symptoms of labial adhesion can be diverse. While some patients are asymptomatic, some present pubic discomfort and dysuria, and in severe cases, urinary retention and subsequent urinary tract infection are observed. In a low oestrogen state, the vaginal mucosa becomes brittle due to the suppression of keratinisation, thus becoming susceptible to inflammation. Furthermore, the production of glycogen, a nutrient source for Döderlein bacillus related to intravaginal self-cleaning action, is reduced, and the vaginal self-cleaning action is considered relatively suppressed [3]. This makes inflammation more likely to occur, causing labial adhesions. In the present case, because the patient underwent hysterectomy at the age of 33 years, we did perform genital tract imaging. However, we could not identify the external urethral meatus due to the adhesion, but additional urinary/genital tract imaging should be planned before any surgical approach if possible.

Recurrence is often a problem in the treatment of labial adhesions, and its recurrence rate after surgical or manual procedure is 14%–20% [4]. If the bilateral labia majora are extensively fused and form large ulcers due to delamination, stenosis of the vaginal orifice can occur due to the temporal contraction of the covering epithelium merely by stitching alone. Such stricture and genital scarring can be prevented by covering the area with a full-thickness skin flap that contains a separate blood supply [5]. However, a possible alternative method would involve several releasing incisions closed with Z-plasty techniques, but this would still result in a scarred vulva and clitoral area. It would also be a cosmetic problem for patients [6]. Although there are reports of successful prevention of labial adhesion by the application of oestrogen ointment in children and postmenopausal women [1,7], it is only available in some countries. In mild cases, management includes the application of topical oestrogen with or without topical steroids. If a response to topical therapy is not noted, surgical separation under anaesthesia should be performed [8].

Reconstruction using the vulvoperineal flap with a superficial external pudendal artery perforator facilitates prevention of recurrence even in an environment using tissue that is not easily affected by oestrogen. This is possibly the greatest advantage of this approach. The main drawback of this method is the potential complications such as flap ischaemia, wound-healing complications, and infection. In addition, it is more invasive with a higher risk of complications than simple sharp dissection and manual separation.

## Conclusion

Here, we report the case of a postmenopausal woman who required reconstruction for severe labial adhesion using a vulvoperineal flap, with the superficial external pudendal artery perforator as the pedicle. In cases with extensive adhesion and repeated recurrence and because erosion formation due to exfoliation becomes more common, reconstruction with a vulvoperineal flap may be preferable over manual separation, sharp surgical dissection, and application of oestrogen ointment. In addition, reconstruction with tissue embryologically unaffected by oestrogen could possibly reduce the rate of recurrence. With an aging population, the number of patients with labial adhesion will possibly increase in the future.
